# INNOVATIONS IN REMOTE SUPPORT FOR DEMENTIA FAMILY CAREGIVERS

**DOI:** 10.1093/geroni/igac059.1545

**Published:** 2022-12-20

**Authors:** Kylie Meyer, Lyndsey Miller, Jeffrey Kaye

## Abstract

Remote delivery of dementia caregiver interventions can decrease delivery costs, and make it more feasible to provide evidence-based interventions to caregivers across the country. As the science behind remote delivery develops, new technologies and their applications can ensure preservation of important intervention components and principles, as well as novel forms of data collection. In this symposium, investigators will present on studies that demonstrate how technology can be used to improve delivery and assessment of remote caregiver interventions. Walter Dawson, D.Phil, will share findings from the Support via Technology: Living and Learning with Advancing Alzheimer’s disease and related dementia (STELLA) intervention. Using secondary data collected via weekly survey, he examined the association between costs of care and behavioral symptoms of dementia. Next, Allison Gibson, PhD, MSW, will present results from focus groups about caregivers’ experiences of the Harmony at HOME (H@H), a telehealth intervention to improve person-environment fit and limit behavioral symptoms of dementia. Kylie Meyer, PhD, will present results from the Learning Skills Together intervention, which uses teleconferencing to teach family caregivers how to provide complex care tasks while adhering to self-efficacy theory. Lastly, Shirin Hiatt, MPH, MS, RN, will present findings from the REmote Assessment and Dynamic Response (READyR) study, which tests the application of remote monitoring technology to assess adherence to value-based care (e.g., autonomy) among spousal family care partners. Each study was supported by the Emory University Roybal Center for Dementia Caregiving Mastery or Oregon Roybal Center for Care Support Translational Research Advantaged by Integrating Technology.

